# Spondylarthritis presenting with an allergic immediate systemic reaction to adalimumab in a woman: a case report

**DOI:** 10.1186/1752-1947-5-155

**Published:** 2011-04-19

**Authors:** Maurizio Benucci, Mariangela Manfredi, Sergio Testi, Maria L Iorno, Maurizio Valentini, Francesca Soldaini, Paolo Campi

**Affiliations:** 1 Rheumatology Unit, Ospedale S. Giovanni di Dio, Via di Torregalli 3, I-50143 Firenze, Italy; 2 Immunology and Allergology Laboratory Unit, Ospedale S. Giovanni di Dio, Via di Torregalli 3, I-50143 Firenze, Italy; 3 Allergy and Clinical Immunology Unit, Ospedale S. Giovanni di Dio, Via di Torregalli 3, I-50143 Firenze, Italy

## Abstract

**Introduction:**

The efficacy of adalimumab, a fully human anti-tumor necrosis factor α recombinant antibody, has dramatically improved the quality of life of patients with rheumatoid and psoriatic arthritis and Crohn's disease. Because it is fully human, one should not expect immune reactions to this molecule. Adverse reactions to adalimumab are limited mainly to injection site reactions and are very common. Immediate systemic reactions are rarely reported.

**Case presentation:**

We report the case of a 61-year-old Caucasian woman who was treated with adalimumab for spondylarthritis and developed injection site reactions after the sixth dose. After a two-month suspension, she recommenced therapy and experienced two systemic reactions. The first occurred after one hour with itching of the palms and soles and angioedema of the tongue and lips. Thirty minutes after the next dose the patient had itching of the palms and soles with diffusion to her whole body, angioedema of the lips, dizziness and visual disturbances. A skin-prick test and intra-dermal tests with adalimumab gave strong positive results at the immediate reading. However, serum-specific immunoglobulin E (IgE) to adalimumab were not detectable by using Phadia solid phase, especially harvested for this case, in collaboration with our Immunology and Allergy Laboratory Unit. Her total IgE concentration was 6.4 kU/L.

**Conclusion:**

We describe what is, to the best of our knowledge, the first reported case of immediate systemic reaction to adalimumab studied with a skin test giving positive results and a serum-specific IgE assay giving negative results. The mechanism of the reaction must be immunologic but not IgE-mediated.

## Introduction

Adalimumab (Humira; Abbott Laboratories, Abbott Park, IL, USA) is a fully human recombinant immunoglobulin G1 (IgG1) monoclonal antibody with specificity for human tumor necrosis factor α (TNF-α). Adalimumab binds to soluble and membrane-bound TNF-α, leading to the blockade of activity of TNF. Apoptosis of cells with membrane-bound TNF occurs. Adalimumab was initially approved for the treatment of rheumatoid arthritis. It was subsequently approved for treatment of psoriatic arthritis, ankylosing spondylitis, juvenile idiopathic arthritis, plaque psoriasis, Crohn's disease and uveitis. The recommended dosage for adults with spondylarthritis is a subcutaneous dose of 40 mg every other week.

The most frequent adverse reactions to adalimumab are injection site reactions. They occur in 6.6% to 15.3% of the patients treated [1-3]. These reactions appear within one to 24 hours at the site of subcutaneous administration and consist of erythema, edema and itching. They peak at 48 hours and last for three to five days. Usually, they occur in the first to second month of therapy and fade over time. The adverse reactions rarely require cessation of treatment, although they are very troublesome.

Immediate systemic reactions to adalimumab have been reported in the literature only in the past three years by six authors [4-9]. Only Rodrìguez-Jiménez *et al*. [9] performed a skin-prick test with a positive result; a desensitization protocol was applied with success. However, no serologic study has been performed with the aim of ascertaining whether the reaction was IgE-mediated.

Here we describe the first case of an immediate systemic adverse reaction to adalimumab with positive skin tests and a serum-specific IgE assay giving negative results.

## Case presentation

We describe the case of a 61-year-old Italian Caucasian woman who did not smoke or drink. Her medical history included hypertension and hypothyroidism after undergoing surgery for thyroid nodulosis, for which she was treated with valsartan, levothyroxine, atenolol and chlorthalidone.

The patient was referred to our Outpatient Clinic for Biological Therapy in the Hospital San Giovanni di Dio in Florence, Italy. In the weeks before referral, her symptoms had worsened, with the appearance of back pain and progressive limitation of mobility. Her history included repeated flares of sciatica, talalgia and trochanteritis. Her modified Schober test result was 15 cm to 18 cm.

An X-ray and a magnetic resonance imaging scan showed sacroiliitis. A diagnosis of spondylarthritis was made. The patient was started on therapy with sulfasalazine 2 g/day and deflazacort 7.5 mg/day for three months, without improvement of her symptoms. In September 2007, because of elevated scores (>30) on both the Bath Ankylosing Spondylitis Functional Index (BASFI) and the Bath Ankylosing Spondylitis Disease Activity Index (BASDAI) scales, the patient was started on biological therapy with adalimumab 40 mg every other week.

After the sixth dose of adalimumab, an injection site reaction occurred after 12 hours, with erythema, edema and itching over a zone 5 cm to 6 cm in diameter. The reaction peaked at 24 to 48 hours and lasted three to four days. Prophylaxis with oral anti-histamines had little efficacy. The clinical efficacy of the drug at that time was satisfactory (BASFI and BASDAI scores both less than 10). We performed prick, intra-dermal and patch tests following the procedure previously described [10].

A commercial preparation of Humira was used which contains, in 0.8 mL of distilled water, adalimumab 40 mg, which is a concentration of 50 mg/mL; mannitol, 9.6 mg; and polysorbate 80, 0.8 mg. For the prick and patch test and for the intra-dermal test with late reading, we used the undiluted drug (50 mg/mL). For the intra-dermal test at immediate reading, we used a concentration of 5 mg/mL. These concentrations proved to be non-irritating in 10 control subjects never treated with adalimumab [11].

For the prick test, a reading was taken after 20 minutes; for the intra-dermal test, readings were taken after 15 minutes and 24 hours; and for the patch test, readings were taken after 48 and 72 hours. Prick and intra-dermal tests with adalimumab showed negative results at the immediate reading; however, after 24 hours, an itchy, red papule 7 mm in diameter, surrounded by an erythema of 15 mm, occurred at the site of the intra-dermal test and lasted five days. The patch test with the undiluted drug was negative, as was an intra-dermal test with mannitol (18 mg/mL).

Adalimumab was withheld for two months. In this period, she received leflunomide. The first two doses after the pause yielded an injection site reaction as previously. After the third dose, the patient experienced, after one hour, a systemic reaction that manifested as itching of the palms and soles and angioedema of the tongue and lips. After the following dose, after 30 minutes, a generalized itching occurred, with lip angioedema, dizziness and visual disturbances. Her symptoms resolved with the administration of intravenous corticosteroids and oral anti-histamine, within one hour in the first case and within two hours in the second case. She also reported decreased efficacy after resuming treatment with the drug. It should also be noted that she had stopped oral corticosteroids at that time, but her BASFI and BASDAI values were both >30.

We repeated the skin tests after the systemic reactions, with strong positive results at the immediate reading: The skin-prick test yielded a wheal 6 mm in diameter and a flare of 15 mm, and the intra-dermal test was positive with 5 mm of wheal and 6 mm of flare until the dilution of 0.005 mg/mL, that is, diluted 10,000-fold. The intra-dermal test was negative at the late reading.

An ImmunoCAP, especially harvested by Phadia, Uppsala, Sweden in collaboration with our Laboratory of Immunology and Allergy, was used to assay serum-specific IgE to adalimumab. A commercial Phadia ImmunoCAP was used for the assay of total IgE and specific IgE to common inhalant allergens (Phadiatop).

*In vitro *tests were not able to demonstrate serum-specific IgE to adalimumab by means of Phadia ImmunoCAP: The result was 0.01 kUA/L. Total IgE was 6.4 kU/L. An atopic status was excluded by history and also by a negative Phadiatop.

## Discussion

We previously described, for the first time, an allergologic study of two patients with injection site reactions to adalimumab. Intra-dermal skin tests with adalimumab were positive at the late reading (24 hours), and patch tests were negative [11]. Up to the present time, we have studied three more patients: two of them showed a positive intra-dermal test at the immediate reading (15 minutes), and one had a positive intra-dermal test at both immediate and late readings. Only one other study exists of two patients with injection site reactions who showed positive intra-dermal tests at the immediate reading and leukocyte histamine release with adalimumab [12]. However, for the intra-dermal test, Paltiel *et al*. [12] used the non-diluted drug at a concentration of 50 mg/mL and tested this concentration in only one control patient. In our experience, the concentration of 50 mg/mL was positive in one of four never-treated control subjects; then, for the intra-dermal test, we used a concentration of 5 mg/mL, which was not irritating in 10 never-treated control subjects.

For the skin prick test, the undiluted drug (50 mg/mL) was not irritating [11]. Adverse reactions to biologic agents have been categorized into five types [13,14]. Adverse reactions of type β, that is, hypersensitivity reactions, are mediated by an immune mechanism, mainly type I (specific IgE), type III (specific IgG) or type IV (lymphocytes) following the Gell and Coombs classifications.

The immunologic mechanism underlying the systemic reactions of our patient seems to be mediated by specific IgE. The presence of specific IgE must be suspected not only because of the types of symptoms (itching, angioedema, dizziness and possible hypotension, as indicated by the visual disturbances, and their occurrence within one hour of administration) but also because the skin tests at immediate reading were strongly positive, as was the prick test, which is less sensitive than the intra-dermal test. The other four patients who had injection site reactions to adalimumab, that is, a less severe reaction, showed positive intra-dermal tests but negative skin prick tests. This would indicate a lower level of specific antibodies.

However, we could not demonstrate the presence of serum-specific IgE in any of these patients, even in those with a positive intra-dermal test at the immediate reading. We also excluded the responsibility of excipients, because the skin test with mannitol was negative. These results suggest that another antibody could be responsible for both the adverse reactions and the positive skin tests.

Non-IgE-mediated anaphylaxis has been described in mice, involving specific IgG, FcγRIII, macrophages, and PAF - platelet activating factor - in cases of repeated exposure to large quantities of antigen [15]. It could be speculated that a similar mechanism was underlying the reactions of our patient. With the aim of ascertaining whether mastocytes are involved in these reactions, it would be useful to assay the serum tryptase in the acute phase of the reaction. In our patient, it was not possible, because the reaction occurred while the patient was at home.

## Conclusion

We describe what is, to the best of our knowledge, the first case of an immediate systemic reaction to adalimumab with positive skin tests and negative research into serum-specific IgE, despite a comprehensive serologic study. The finding of positive skin tests suggests that an immunologic mechanism is responsible for this adverse reaction; however, serum-specific IgE to adalimumab could not be demonstrated by means of Phadia ImmunoCAP. Therefore, the exact nature of this immunologic mechanism and the antibody responsible for it remain to be elucidated.

The clinical consequences of this finding are limited, because the cases of adverse systemic reactions to adalimumab are rare. However, the conceptual implications of this case are highly relevant, because we demonstrated an adverse immune-mediated reaction to a fully human recombinant biologic response modifier, not only in this case but also in some cases of injection site reactions to adalimumab.

## Competing interests

The authors declare that they have no competing interests.

## Consent

Written informed consent was obtained from the patient for publication of this case report and any accompanying images. A copy of the written consent is available for review by the Editor-in-Chief of this journal.

## Authors' contributions

MB reported the case to PC and described the clinical picture and the patient's adverse reaction. ST, MLI and PC did the skin tests. PC was a major contributor in writing the manuscript. MM, MV and FS did the *in vitro *tests. All authors read and approved the final manuscript.
